# A Validated HPLC–Diode Array Detection Method for Therapeutic Drug Monitoring of Thiopurines in Pediatric Patients: From Bench to Bedside

**DOI:** 10.3390/metabo12121173

**Published:** 2022-11-24

**Authors:** Martina Franzin, Debora Curci, Marianna Lucafò, Matteo Bramuzzo, Marco Rabusin, Antonella Fabretto, Riccardo Addobbati, Gabriele Stocco, Giuliana Decorti

**Affiliations:** 1 Institute for Maternal and Child Health, IRCCS “Burlo Garofolo”, 34137 Trieste, Italy; 2 Clinical Department of Medical, Surgical and Health Sciences, University of Trieste, I-34149 Trieste, Italy

**Keywords:** thiopurines, therapeutic drug monitoring, pediatric patients

## Abstract

Thiopurine drugs are part of the therapeutic armamentarium for pediatric patients suffering from inflammatory bowel disease (IBD) and acute lymphoblastic leukemia (ALL). The therapeutic drug monitoring of these drugs, consisting of measurements of the thiopurine metabolites thioguanine nucleotides (TGN) and methylmercaptopurine nucleotides (MMPN) are used to optimize the effectiveness of treatment and prevent adverse effects. In this context, we developed and validated a high-performance liquid chromatography—diode array detection (HPLC–DAD) method for the simultaneous quantification of thiopurine metabolites according to the most recent International Council for Harmonisation (ICH) guidelines. The calibration curves were built in the clinically relevant range of concentrations for TGN of 300–12,000 nM and for MMPN of 3000–60,000 nM. The limit of detection and the lower limit of quantification were 100 and 300 nM for TGN and 900 and 3000 nM for MMPN, respectively. The percentage of inter-day accuracy and precision (CV%) varied between 85 and 104% and 1.6 and 13.8%. Stability was demonstrated for both of the metabolites for at least 50 days at −20 °C. The proposed HPLC–DAD method showed an appropriate selectivity, specificity, linearity, accuracy, precision and good applicability to samples from patients with IBD and ALL undergoing thiopurine treatment.

## 1. Introduction

Thiopurines, such as mercaptopurine (MP), its prodrug azathioprine (AZA) and thioguanine (TG), are part of the therapeutic armamentarium for the treatment of several pediatric diseases including inflammatory bowel disease (IBD) and acute lymphoblastic leukemia (ALL) [1,2,3]. In particular, AZA and MP, alone or in combination with biological agents, are used in pediatric patients suffering from IBD, Crohn’s disease (CD) or ulcerative colitis (UC) at the recommended daily dose of 2.5 and 1.5 mg/kg to maintain remission [1,2]. Instead, the administration of MP and TG, combined with other drugs such as methotrexate, is a mainstay in the protocols of maintenance therapy for pediatric ALL patients [3].

**Figure 1:**
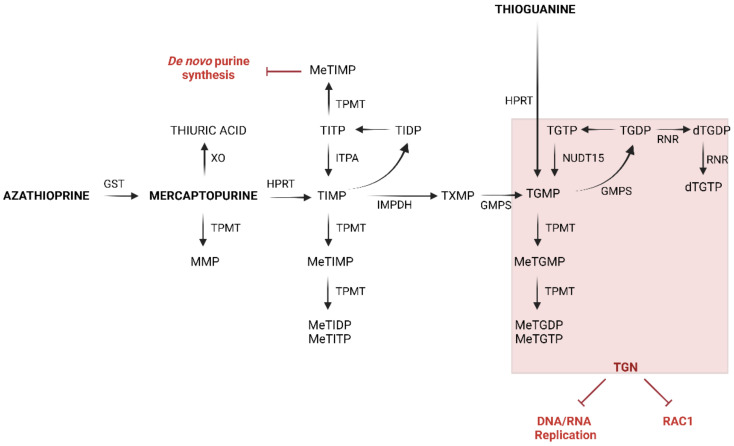
Thiopurine pathways. Compounds: MMP: methylmercaptopurine, TIMP: thioinosine monophosphate, TIDP: thioinosine diphosphate, TITP: thioinosine triphosphate, TXMP: thioxanthosine monophosphate, TGMP: thioguanosine monophosphate, TGDP: thioguanosine diphosphate, TGTP: thioguanosine triphosphate. Enzymes: GST: glutathione S-transferase, GMPS: guanosine monophosphate synthetase, HPRT: hypoxanthine-guanine phosphoribosyltransferase, IMPDH: inosine monophosphate dehydrogenase, ITPA: inosine triphosphate pyrophosphatase, NUDT15: nudix type 15—nucleoside diphosphate-linked moiety X-type motif 15, XO: xanthine oxidase, TPMT: thiopurine S-methyltransferase, RAC1: Ras-related C3 botulinum toxin substrate 1.

Since these agents are purine analogous, they act as antimetabolites exerting lympholytic effects via a complex pathway (Figure 1), leading to the formation of thiopurine metabolites: the thioguanine nucleotides (TGN) responsible for cytotoxicity through nucleic acid damage, cell-cycle arrest and apoptosis, and the methylmercaptopurine nucleotides (MMPN), which are associated with hepatotoxicity and whose generation competes with the production of the former metabolites [4].

Therapeutic drug monitoring (TDM) enables to optimize the management of patients undergoing treatment, increasing drug efficacy and preventing adverse effects [5]. TDM is particularly useful in the pediatric population; indeed, children’s responses to drugs are different from those of adults due to differences in pharmacokinetics and pharmacodynamics [6,7].

Although the efficacy of thiopurines has been largely demonstrated, up to 30% of patients discontinue treatment because of unsatisfying therapeutic effects and toxicity [8]. Monitoring the levels of thiopurine metabolites has been shown to enhance the frequency of clinical remission and reduce adverse reactions [9,10]. In IBD, the therapeutic levels are considered to be 235–450 pmol/8 × 10^8^ red blood cells (RBCs) for TGN and <5700 pmol/8 × 10^8^ RBCs for MMPN [8].

In this context, starting from the original Dervieux-Bolieu method [11], we readapted and validated a high-performance liquid chromatography coupled to diode array detection (HPLC–DAD) method, for the simultaneous quantification of thiopurine metabolites according to International Council for Harmonisation (ICH) guidelines (ICH guideline M10 on bioanalytical method validation and ICH guideline Q2(R2) on validation of analytical procedures), and we tested its applicability on cohorts of pediatric IBD and ALL patients.

## 2. Materials and Methods

### 2.1. Chemicals and Materials

Methylmercaptopurine (MMP) was purchased from Alfa Aesar (Kandel, Germany). TG, the internal standard (IS) bromouracile (BU), DL-dithiothreitol (DTT), perchloric acid (HClO_4_) (70%), potassium dihydrogenphosphate (KH_2_PO_4_), phosphoric acid (H_3_PO_4_) and methanol were purchased from Merck (Milan, Italy). The filter paper (particle retention: 10–20 µm) was purchased from VWR (Milan, Italy). All of the solutions were prepared with HPLC grade water obtained from a Milli-Q Plus water purification system.

### 2.2. Stock and Working Solutions

The stock solutions of 100 mM TG and 100 mM MMP were prepared in DMSO and stored at −20 °C. On the day of the analysis, working solutions of 20 µM TG and 100 µM MMP were prepared fresh from stock solutions by diluting, respectively, the analytes at 1:5000 and 1:1000 in HPLC grade water. Different working solutions were used for the construction of calibration curves and for the preparation of quality controls (QCs).

On the day of the analysis, the IS solution of 314 µM BU was prepared fresh in HPLC grade water.

A stock solution of 500 mg/mL DTT was prepared in HPLC grade water and stored at −20 °C.

### 2.3. Calibration and Quality Control Samples

The calibration standards (CALs) and quality controls (QCs) samples were prepared by spiking the RBCs lysate pool with different amounts of the working solutions of 20 µM TG and 100 µM MMP. The working solutions were prepared separately for the construction of calibration curves and for the preparation of QCs to avoid biased estimations.

The calibration curves were built from 300 to 12,000 nM for TGN and from 3000 to 60,000 nM for MMPN on the basis of the therapeutic range that was expected [8]. Taking into account the calibration range and the ICH guidelines, the chosen QCs were the lower limit of quantification (LLOQ) (TGN: 300 nM; MMPN: 3000 nM), within about 3 times of the LLOQ (low QC-LQC) (TGN: 800 nM; MMPN: 8000 nM), around 30–50% of the calibration curve range (medium QC-MQC) (TGN: 4000 nM; MMPN: 20,000 nM) and at 75% of the ULOQ (high QC-HQC) (TGN: 9000 nM; MMPN: 45,000 nM).

The IS BU was added at the beginning of the extraction procedure in all of the CALs and QCs, and the preparation was the same that is reported here for the sample preparation.

### 2.4. Blood Sample Collection

The peripheral blood (2–3 mL) from healthy donors (*n* = 6) and pediatric IBD (*n* = 10) and ALL patients (*n* = 6) was collected in sodium heparin-containing tubes with 10 µL of 100 mg/mL DTT. Samples from routine analyses left over at the Advanced Translational Diagnostics Laboratory were used to test the applicability of the proposed validated method. After centrifugation at 800× *g* for 10 minutes at 4 °C, the plasma and buffy coats were discarded, and the RBCs were washed twice with sodium chloride 0.9%. Purified RBCs from pediatric patients were counted for normalizing the concentration of thiopurine metabolites obtained (nM) in the commonly used unit of measure pmol/8 × 10^8^ RBCs. Subsequently, RBCs were lysed adding 1 part of MilliQ water to 2 parts of RBCs and they were vortexed, aliquoted in 500 µL and stored at −20 °C for a maximum of 2 weeks until the day of sample preparation.

### 2.5. Sample Preparation

RBCs lysates, CALs or QCs were thawed and brought to room temperature, then, 10 µL of 500 mg/mL DTT were added to the samples. The precipitation of the proteins was achieved by adding 50 µL of HClO_4_ and vortexing for 30 s. After centrifugation at 16,000× *g* for 15 minutes at 20 °C, the supernatant was transferred into a new tube and heated for 1 hour at 95 °C to allow the process of hot acid hydrolysis to occur through which TGN and MMPN are converted, respectively, in TG and an MMP derivative, as previously reported [12]. The samples were then cooled down for 20 minutes at −20 °C to stop the reaction, and after reaching room temperature, 100 µL of the sample was injected in the instrument.

### 2.6. Instrumentation

The chromatographic system consisted of an Agilent 1260 Infinity II (Agilent Technologies, Milan, Italy) coupled with a Diode Array Detector WR (Agilent Technologies, Milan, Italy) that operated in a wide wavelength range from 190 to 950 nm. OmniSpher 5 C18 250 *×* 4.6 mm, 5 µm (Agilent Technologies, Milan, Italy) column equipped with a guard cartridge (ChromSep) Pursuit PAH, 200Å, 3 *×* 10 mm (Agilent Technologies, Milan, Italy) was used for the analysis.

A chromatographic separation was obtained after elution of mobile phase A (0.02 M potassium dihydrogenophosphate buffer titrated to the desired pH = 3.5 with phosphoric acid) and mobile phase B (methanol) in the gradient mode that is described below (Table 1) for a total run of 18 minutes at a flow rate of 1.1 mL/min. The injection volume was 100 µL.

The absorbances set were 280, 304 and 341 nm for the detection and quantification, respectively, of BU, MMPN and TGN.

### 2.7. Data Analysis

The chromatograms were recorded and analyzed with the software Agilent OpenLab Software version 2.0 (Agilent Technologies, Milan, Italy). The interquartile range (IQR) was represented as the interval between the first and the third quartile.

## 3. Results

### 3.1. Method Development

**Figure 2:**
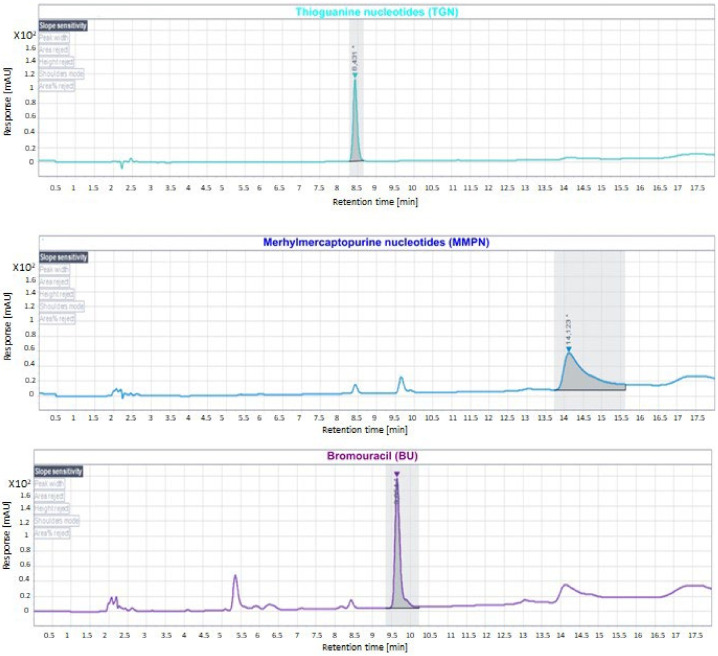
Chromatogram showing the retention times in minutes of the analytes TGN and MMPN (green and blue lines) and of IS (purple line). * indicates the retention times.

As shown in Figure 2, the retention times of TGN and MMPN were 8.4 and 14 minutes, respectively, and the retention time of the IS BU was 9 minutes.

### 3.2. Method Validation

#### 3.2.1. Selectivity and Specificity

The selectivity of the analytical method was evaluated by analyzing six individual blank samples, which are referred as matrix samples without analyte or IS. The responses of the blanks detected at the retention times of analytes were not more than 20% of those of analytes at LLOQ; the response of the blanks detected at the retention time of IS was not more than 5% of that of IS.

Moreover, a lack of significant response of interfering components different from the analytes and IS in the chromatograms has been ascertained for the assessment of specificity.

#### 3.2.2. Linearity

The linearity was assessed through the construction of calibration curves in duplicate in three different analytic runs. In particular, the concentrations of the CALs were 300, 600, 1000, 2000, 3000, 6000 and 12,000 nM for TGN (*n* = 7) and 3000, 5000, 10,000, 15,000, 30,000 and 60,000 nM (*n* = 6) for MMPN. The calibration curves were built, plotting the areas of each CALs corrected with the IS area versus the theoretical values. No statistical weight was applied to the calibration curves. Accuracy and precision, represented by the coefficient of variation (CV%), were also evaluated within each run (Table 2 and Table 3) and in different runs (Table 4 and Table 5).

#### 3.2.3. Sensitivity

The limit of detection (LOD), referred as the lowest concentration of analytes that are able to be detected by the method, and the LLOQ, referred as the lowest concentration of analytes that are able to be quantified by the method, were determined based on signal-to-noise approach, according to ICH guidelines, and based on the standard deviation of the y-intercept and the slope of the calibration curve. Both approaches gave the same results: in particular, the LOD and LLOQ values were 100 and 300 nM for TGN and 900 and 3000 nM for MMPN, respectively.

Furthermore, LLOQ and LOD values were confirmed by spiking CAL1 and further dilutions. In particular, diluting CAL1 resulted in loss of accuracy, therefore, CAL1 can be defined as LLOQ. Instead, 100 nM TG and 900 nM MMPN resulted also the last concentrations obtained with dilution at which there was a distinguishable signal.

#### 3.2.4. Accuracy and Precision

The accuracy and precision, represented as CV (%), within each run (Table 6 and Table 7) and in different runs (Table 8 and Table 9) were assessed on the QCs. The concentration was calculated on the basis of the calibration curve. In particular, the percentage of the intra-day and inter-day accuracy was in the accepted range (100 ± 20% for LLOQ and 100 ± 15% for the other QCs). Additionally, the intra-day and inter-day CV (%) did not exceed the threshold value of 15%.

#### 3.2.5. Carryover

**Figure 3:**
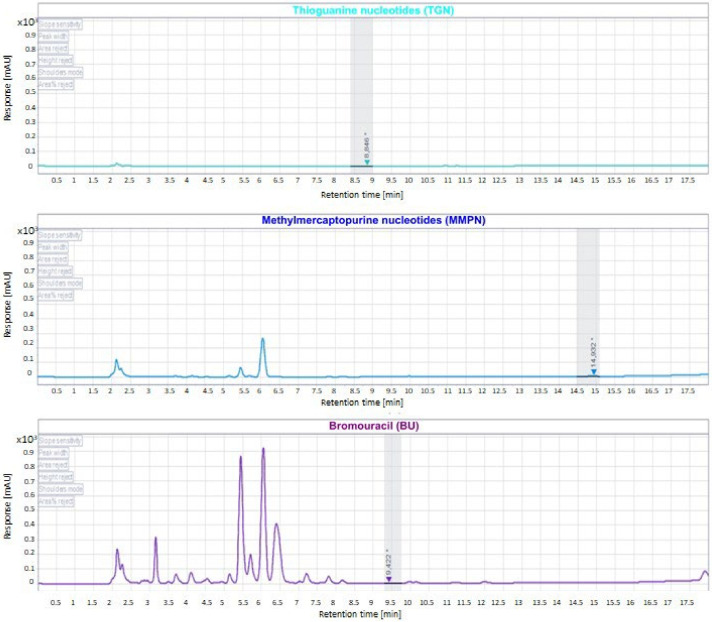
Chromatogram showing blank sample after the injection of CAL with the highest concentration. * indicates the retention times.

The carryover was evaluated by injecting blank samples after the CAL with the highest concentration. Figure 3 shows that the response derived from the blank at the retention time of interest was not greater than 20% of the analytes and 5% of IS.

#### 3.2.6. Matrix Effect

The alteration of the analyte response because of the interfering components in the biological matrix, the so-called matrix effect, was evaluated by analyzing in triplicate the LQC and HQC which were prepared from six different biological matrices and testing the accuracy and precision of these samples. The percentage of accuracy did not vary more than ±15% and the CV (%) did not exceed 15%, threshold values recommended by ICH guidelines, suggesting that there is no matrix effect influencing the performance of the analytical method.

#### 3.2.7. Stability

Since the working solutions were prepared fresh on the day of the analysis, only the stability of the stocking solution was evaluated after 30 and 50 days by testing the accuracy and precision of the QCs. Moreover, also stability of the compounds in the biological matrix was assessed for the QCs that were frozen and analyzed after 30 and 50 days. The TG and MMP were both stable in the stock solution in DMSO and in the biological matrix for 50 days at −20 °C (Table 10 and Table 11).

### 3.3. Application to Samples

**Figure 4:**
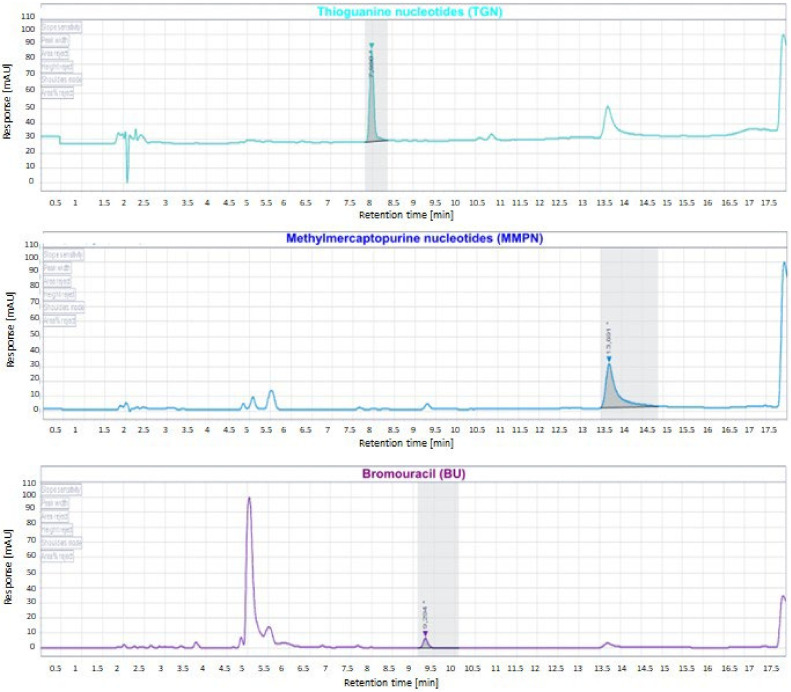
Chromatogram derived from the injection of a real sample containing TGN and MMPN (517.94 and 12,255.06 pmol/8 × 10^8^ RBC, respectively). * indicates the retention times.

The method was applied for the quantification of TGN and MMPN in the patients suffering from IBD and ALL. An example of the chromatogram derived from a real sample is reported in Figure 4.

The results, expressed as pmol/8 × 10^8^ RBC, unit of measure commonly used for thiopurine metabolites quantification in the literature [8], were in line with previously reported data confirming the reproducibility of this new validated method in patient’s cohort of ALL and IBD [4,13]. Results of measurements are summarized in Table 12.

## 4. Discussion

Despite the proven efficacy of thiopurines, a subset of patients undergoing treatment lose response to these drugs and develops adverse reactions [8,10]. In particular, the interindividual variability has been associated with genetic polymorphisms of the genes involved in the thiopurine pathway, affecting the activity of enzymes and the concentrations of the drug metabolites [4]. Therefore, testing the genotype or activity of the enzymes or monitoring the concentration of thiopurine metabolites in RBCs has become an important tool for helping clinicians to decide the most appropriate therapeutic option [8].

Several studies have assessed the correlation between the clinical response to thiopurines, and the levels of the drug metabolites and the therapeutic levels of TGN and MMPN have been proposed. In IBD, values of 235–450 pmol/8 × 10^8^ RBC and MMPN < 5700 pmol/8 × 10^8^ RBC are considered to be in the therapeutic range [8]. Monitoring these parameters is particularly important in IBD and ALL pediatric patients who are being treated with these drugs. Of note, the pediatric population responds differently to medications in comparison to adults due to the pharmacokinetic and pharmacodynamic differences [6,7].

In order to provide reliable information to clinicians, the analytical methods that allow us to quantify the compounds of interest in a biological matrix should be developed and validated. Several techniques were used to perform the quantification of thiopurine metabolites in RBCs [12,14,15]. In particular, liquid chromatography coupled with mass spectrometry (LC-MS) allows for the detection and identification of mono-, di- and triphosphate nucleotides of thioguanosine and methylthioinosine, but it is expensive and uses IS not commercially available; therefore, it is not usually implemented in diagnostics [14]. Instead, HPLC coupled with UV or DAD detection is the most used because it is relatively cheap, and once the method has been validated, can be easily implemented for diagnostics, although it allows only the discrimination between TGN and MMPN.

Dervieux and Bolieu were among the first to set-up a fast and easy method to quantify TGN and MMPN through an HPLC-UV technique using a sample preparation based on hot acid hydrolysis, leading to the detection of the free-base TG and a derivate compound of MMP which is formed after this process [11]. Nevertheless, the manuscript of Dervieux and Bolieu was more focused on the innovative sample preparation than on the validation of the method, according the most up-to-date ICH guidelines, and the authors tested the applicability of the method on a different cohort, in particular, lung or heart/lung transplant patients undergoing AZA therapy [11].

Additionally, Cangemi and colleagues proposed an HPLC method for the quantification of thiopurine metabolites by testing its applicability in a pediatric IBD cohort on two types of biological matrix (RBCs and white blood cells) [12]. However, the guidelines applied for the validation of the method were not updated, and there is no information about ALL patients; moreover, we demonstrated for our method, a higher sensitivity (30 pmol/8 × 10^8^ RBC) for TGN compared to what had been described previously.

Interestingly, since there is an important role for TDM of thiopurines in optimizing the therapy of patients undergoing treatment, another manuscript by Lim and colleagues describing an HPLC-UV method for the quantification of the analytes of interest in a cohort of Malaysian patients suffering from IBD was recently published [16]. In particular, it was successful in measuring the drugs metabolites correlated with clinical scores of disease, except for one analyte, with a suboptimal accuracy and a low extraction efficiency [16].

Since there is no fully standardized diagnostic analytical method for the detection of TGN and MMPN in RBCs, taking into account the sample preparation protocol of Dervieux-Bolieu [11], we developed and validated a HPLC–DAD method that is to be used in the diagnostic routine for the simultaneous quantification of thiopurine metabolites according to the most recent International Council for Harmonisation (ICH) guidelines (ICH guideline M10 on bioanalytical method validation and ICH guideline Q2(R2) on validation of analytical procedures).

The proposed HPLC–DAD method can be easily replicated in other laboratories thanks to the use of common instrumentation and its the fast sample preparation. Moreover, the sensibility and linearity of the method allow for the quantification of the concentrations of thiopurine metabolites in IBD and ALL cohorts of our interest. No interfering compounds, matrix effect and carry over, which could affect negatively the analysis, were detected. The results on accuracy and precision have demonstrated the reliability of the quantification of the analytes. Based on the time that usually passes before performing the analyses, we demonstrated the stability of TG and MMP in the stocking solution and in the biological matrix, which was used for the CAL and QCs, for at least 50 days.

The present study showed some limits. For instance, the peak related to MMPN is characterized by a tailing effect. Moreover, some impurities in the biological matrix were present even if they did not interfere with the quantification of the compounds or influence the specificity of the method.

In conclusion, the described HPLC–DAD method has shown good applicability to pediatric IBD and ALL cohorts, and it could be easily implemented in the diagnostic routine for TDM of pediatric patients undergoing thiopurine treatment.

## Figures and Tables

**Table 1:** Gradient for HPLC–DAD analysis for the quantification of thiopurine metabolites in RBCs lysate.

Min	A (%)	B (%)
0.00	100	0
13.50	85.1	14.9
14.00	82.2	17.8
15.00	82.2	17.8
15.50	100	0
18.00	100	0

**Table 2:** Intra-day accuracy and precision (CV%) referred to CALs of TGN.

Calibration Standard TGN	Theoretical Concentration (nM)	Mean of Calculated Concentration (nM)	Standard Deviation of Calculated Concentration (nM)	Intra-Day Accuracy (%)	Intra-Day Precision (CV%)
1° CALIBRATION CURVE R^2^ = 0.9998 y = 19,128x + 118.62					
CAL1	300	354.75	24.14	81.75	6.81
CAL2	600	637.06	10.15	93.82	1.59
CAL3	1000	996.17	25.09	100.38	2.52
CAL4	2000	1960.21	229.95	101.99	11.73
CAL5	3000	3009.66	115.09	99.68	3.82
CAL6	6000	5885.65	229.91	101.91	3.91
CAL7	12,000	12,056.91	54.62	99.53	0.45
2° CALIBRATION CURVE R^2^ = 0.9995 y = 19,500x + 158.46					
CAL1	300	358.18	0.39	80.61	0.11
CAL2	600	650.69	11.95	91.55	1.84
CAL3	1000	1029.91	46.86	97.01	4.55
CAL4	2000	1991.30	90.18	100.44	4.53
CAL5	3000	2790.85	104.91	106.97	3.76
CAL6	6000	6073.07	151.74	98.78	2.50
CAL7	12,000	12,006.16	40.88	99.95	0.34
3° CALIBRATION CURVE R^2^ = 0.9977 y = 23,470x + 21.831					
CAL1	300	270.05	39.02	109.98	14.45
CAL2	600	649.54	61.32	91.74	9.44
CAL3	1000	866.05	43.92	113.39	5.07
CAL4	2000	1862.86	23.32	106.86	1.25
CAL5	3000	3051.24	42.56	98.29	1.39
CAL6	6000	6402.59	203.32	93.29	3.18
CAL7	12,000	11,797.84	663.86	101.68	5.63

**Table 3:** Intra-day accuracy and precision (CV%) referred to CALs of MMPN.

Calibration Standard MMPN	Theoretical Concentration (nM)	Mean of Calculated Concentration (nM)	Standard Deviation of Calculated Concentration (nM)	Intra-Day Accuracy (%)	Intra-Day Precision (CV%)
1° CALIBRATION CURVE R^2^ = 0.9999 y = 32,320x − 12.029					
CAL1	3000	3273.48	39.18	90.88	1.20
CAL2	5000	5054.33	130.03	98.91	2.57
CAL3	10,000	9761.71	1277.15	102.38	13.08
CAL4	15,000	14,789.21	38.10	101.41	0.26
CAL5	30,000	30,101.20	1579.23	99.66	5.25
CAL6	60,000	60,021.19	2120.93	99.96	3.53
2° CALIBRATION CURVE R^2^ = 0.9995 y = 33,222x + 893.43					
CAL1	3000	3518.60	337.34	82.71	9.59
CAL2	5000	5186.80	91.88	96.26	1.77
CAL3	10,000	9163.05	990.54	108.37	10.81
CAL4	15,000	15,201.29	1040.12	98.66	6.84
CAL5	30,000	29,801.38	618.70	100.66	2.08
CAL6	60,000	60,128.14	299.85	99.79	0.50
3° CALIBRATION CURVE R^2^ = 0.9971 y = 39,182x − 1501.9					
CAL1	3000	2819.19	63.59	106.03	2.26
CAL2	5000	4693.87	617.27	106.12	13.15
CAL3	10,000	8648.54	739.45	113.51	8.55
CAL4	15,000	16,187.43	5.22	92.08	0.03
CAL5	30,000	31,602.78	2559.76	94.66	8.10
CAL6	60,000	59,046.46	7131.64	101.59	12.08

**Table 4:** Inter-day accuracy and precision (CV%) referred to CALs of TGN.

Calibration Standard TGN	Inter-Day Accuracy (%)	Inter-Day Precision (CV%)
CAL1	90.78	15.23
CAL2	92.37	1.17
CAL3	103.60	8.97
CAL4	103.09	3.46
CAL5	101.65	4.74
CAL6	97.99	4.28
CAL7	100.39	1.15

**Table 5:** Inter-day accuracy and precision (CV%) referred to CALs of MMPN.

Calibration Standard MMPN	Inter-Day Accuracy (%)	Inter-Day Precision (CV%)
CAL1	93.21	11.08
CAL2	100.43	5.12
CAL3	108.09	6.06
CAL4	97.38	4.67
CAL5	98.33	3.16
CAL6	100.45	1.00

**Table 6:** Intra-day accuracy and precision (CV%) referred to QCs of TGN.

Quality Controls TGN	1°		2°		3°	
	Intra-Day Accuracy (%)	Intra-Day Precision (CV%)	Intra-Day Accuracy (%)	Intra-Day Precision (CV%)	Intra-Day Accuracy (%)	Intra-Day Precision (CV%)
LLOQ	81.11	5.48	80.04	4.81	93.71	11.15
LQC	93.78	2.62	92.45	5.80	86.73	3.60
MQC	95.36	1.68	98.06	1.83	85.92	6.63
HQC	87.02	1.21	95.41	4.41	89.67	3.53

**Table 7:** Intra-day accuracy and precision (CV%) referred to QCs of MMPN.

Quality Controls MMPN	1°		2°		3°	
	Intra-Day Accuracy (%)	Intra-Day Precision (CV%)	Intra-Day Accuracy (%)	Intra-Day Precision (CV%)	Intra-Day Accuracy (%)	Intra-Day Precision (CV%)
LLOQ	100.44	4.67	98.52	8.19	110.89	3.71
LQC	102.78	7.00	100.62	11.72	89.33	5.78
MQC	98.98	4.01	103.40	5.68	86.48	6.91
HQC	100.12	1.22	98.08	2.71	103.82	5.69

**Table 8:** Inter-day accuracy and precision (CV%) referred to QCs of TGN.

Quality Controls TGN	Inter-Day Accuracy (%)	Inter-Day Precision (CV%)
LLOQ	84.95	6.61
LQC	90.99	3.44
MQC	93.12	5.96
HQC	90.70	3.92

**Table 9:** Inter-day accuracy and precision (CV%) referred to QCs of MMPN.

Quality Controls MMPN	Inter-Day Accuracy (%)	Inter-Day Precision (CV%)
LLOQ	103.29	6.88
LQC	97.58	7.06
MQC	96.29	8.46
HQC	100.67	2.93

**Table 10:** Evaluation of stability of TG and MMP after 30 days, represented as accuracy and precision values, in the biological matrix.

QCs	TG		MMP	
	Accuracy (%)	Precision (CV%)	Accuracy (%)	Precision (CV%)
LLOQ	95.88	11.26	111.49	10.91
LQC	113.47	10.16	100.96	9.91
MQC	109.38	4.01	87.11	4.51
HQC	85.22	0.25	101.12	1.66

**Table 11:** Evaluation of stability of TG and MMP after 50 days, represented as accuracy and precision values, in the biological matrix.

QCs	TG		MMP	
	Accuracy (%)	Precision (CV%)	Accuracy (%)	Precision (CV%)
LLOQ	87.87	10.24	104.00	8.29
LQC	106.63	7.69	94.48	8.57
MQC	119.88	8.41	95.93	8.95
HQC	85.10	1.35	95.83	7.21

**Table 12:** Summary of metabolites’ concentrations (median and interquartile range (IQR)) in inflammatory bowel disease (IBD) and acute lymphoblastic leukemia (ALL) cohorts.

DISEASE TYPE	TGN (pmol/8 × 10^8^ RBC) [IQR]	MMPN (pmol/8 × 10^8^ RBC) [IQR]
IBD	335.94 [165.44–496.80]	1275.36 [413.75–6186.18]
ALL	627.66 [470.78–762.95]	1466.08 [734.95–1933.12]

## Data Availability

The raw data supporting the conclusions of this article will be made available by the authors, without undue reservation.
